# Small Molecule Inhibitors of CRM1

**DOI:** 10.3389/fphar.2020.00625

**Published:** 2020-05-07

**Authors:** Bibiana I. Ferreira, Bastien Cautain, Inês Grenho, Wolfgang Link

**Affiliations:** ^1^Centre for Biomedical Research (CBMR), University of Algarve, Faro, Portugal; ^2^Regenerative Medicine Program, Department of Biomedical Sciences and Medicine, University of Algarve, Faro, Portugal; ^3^Algarve Biomedical Center (ABC), University of Algarve, Faro, Portugal; ^4^Fundacion MEDINA Parque tecnológico ciencias de la salud, Granada, Spain; ^5^Evotec France, Toulouse, France; ^6^Instituto de Investigaciones Biomédicas “Alberto Sols” (CSIC-UAM), Madrid, Spain

**Keywords:** CRM1, nuclear export, natural products (NP), leptomycin B, Selinexor, high content screening (HCS)

## Abstract

The transport through the nuclear pore complex is used by cancer cells to evade tumor-suppressive mechanisms. Several tumor-suppressors have been shown to be excluded from the cell nucleus in cancer cells by the nuclear export receptor CRM1 and abnormal expression of CRM1 is oncogenic. Inhibition of CRM1 has long been postulated as potential approach for the treatment of cancer and to overcome therapy resistance. Furthermore, the nuclear export of viral components mediated by the CRM1 is crucial in various stages of the viral lifecycle and assembly of many viruses from diverse families, including coronavirus. However, the first nuclear export inhibitors failed or never entered into clinical trials. More recently CRM1 reemerged as a cancer target and a successful proof of concept was achieved with the clinical approval of Selinexor. The chemical complexity of natural products is a promising perspective for the discovery of new nuclear export inhibitors with a favorable toxicity profile. Several screening campaigns have been performed and several natural product-based nuclear export inhibitors have been identified. With this review we give an overview over the role of CRM1-mediated nuclear export in cancer and the effort made to identify and develop nuclear export inhibitors in particular from natural sources.

## Introduction

Human diseases often involve alterations in the structure, localization, interactions, and as a consequence, the function of cellular proteins (Hung and Link, 2011). Normal cell physiology requires a tightly regulated, coordinated activity of thousands of proteins, that must be in the right place at the right time. Protein function depends on the subcellular localization as it determines access of the protein to binding partners and enzymes that catalyze post-translational modification and facilitates its contribution to functional networks. For example, transcription factors have to be in the cell nucleus in order to have access to the target gene promoters and to exert their transcriptional activity. With the exception of red blood cells, all eukaryotic cells contain a nucleus which is surrounded by a nuclear envelope made up of two lipid bilayer membranes. The nuclear envelope represents a greater physical barrier than the single lipid bilayer of the plasma membrane. The movement of molecules between the nucleoplasm and the cytoplasm is controlled by nuclear pore complexes (NPCs). The NPC is one of the largest cellular protein complexes and is responsible for the controlled passage of macromolecules into and out of the nucleus. The NPCs are permeable only to small molecules such as salts, nucleotides, small proteins. Proteins over 40 kDa are required to be moved through the nuclear pore complex (NPCs) by soluble nuclear transport receptors (Cautain et al., 2015b). Proteins that enter and exit through the NPC usually contain specific transport signals namely a nuclear localization signal (NLS) or a nuclear export signal (NES). These sequences are recognized by soluble transport receptors of the karyopherin family.

## The CRM1 Export Receptor

**Figure 1:**
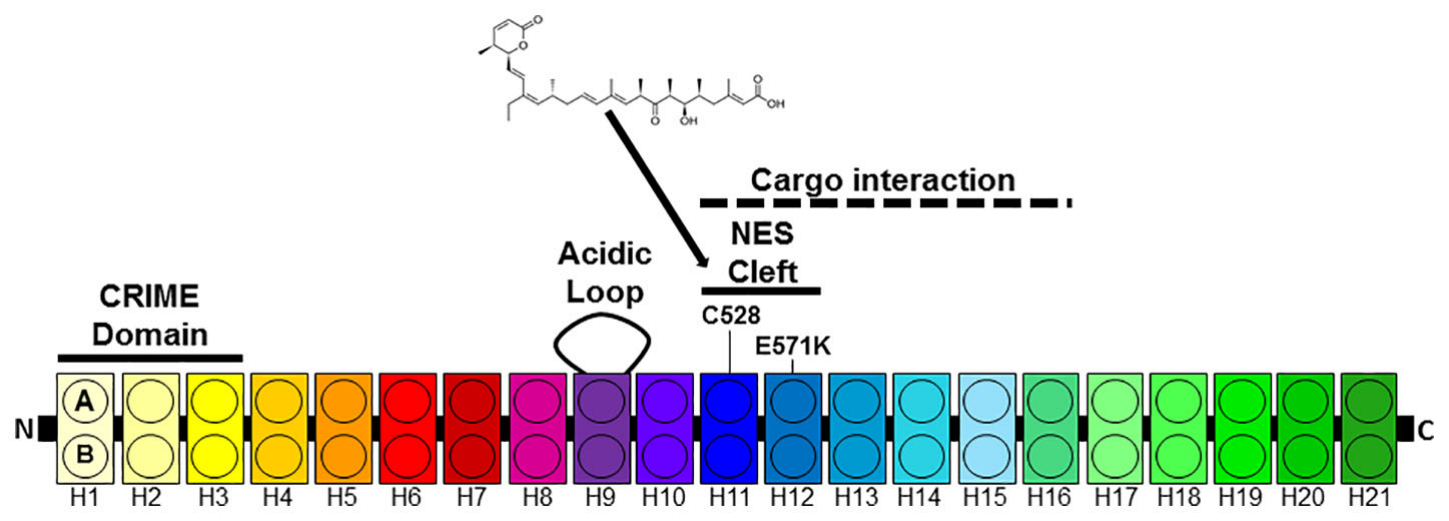
Architecture of CRM1. CRM1 consists of 21 HEAT repeats each composed of two anti-parallel helices **(A**, **B)** connected by a linker loop. The N-terminal CRIME domain and the acidic loop contribute to RanGTP binding. The A helices of HEAT tandem 11 and 12 form the NES cleft. HEAT repeats 11–16 are involved in cargo e.g. Snurportin1 (Spn1) binding.

The best studied export protein is chromosome region maintenance 1 (CRM1 also known as XPO1 or exportin 1) (Fornerod et al., 1997; Fukuda et al., 1997; Ossareh-Nazari et al., 1997; Kudo et al., 1998). CRM1 is expressed in all eukaryotic cells. CRM1 belongs to the karyopherin-β family of transport receptors and mediates the nuclear exports of proteins that contain leucine-rich NESs (Fukuda et al., 1997). Over 200 proteins have been verified as cargoes of CRM1. Ran-GTP binds to CRM1 in the nucleus causing an increased affinity to the NES containing cargo protein and a complex between Ran, CRM1 and the cargo forms. This complex is exported through the NPC into the cytoplasm where Ran-GTP is converted into Ran-GDP and the complex dissociates. Human CRM1 consists of 1071 amino-acid residues and contains several functional regions (**Figure 1**). CRM1 is made up of 21 tandemly repeated, 37–47 amino acid long modules called HEAT protein domains (Dong et al., 2009a; Dong et al., 2009b; Monecke et al., 2009). Each repeat forms a hairpin of two helices called A and B helices building a ring-shaped structure. While the outer surface of the ring comprise the A helices, the B helices form the inner surface (Güttler et al., 2010; Koyama and Matsuura, 2010; Dian et al., 2013; Monecke et al., 2013; Saito and Matsuura, 2013). The NES binding cleft is formed by HEAT repeats 11 and 12 at the outer surface of CRM1. The N-terminal CRIME domain shares sequence homology with importin-β and is involved in binding to RanGTP. The acidic loop within HEAT-repeat 9 also contributes to RanGTP binding and is thought to inhibit the interaction with NES in the absence of RanGTP. While the central part of CRM1 is involved the interaction with NES, the C-terminal end of CRM1 is thought to modulate its affinity to NES (Dong et al., 2009a; Dian et al., 2013).

### The Role of CRM1 in Cancer

CRM1 is responsible for the nuclear export of a large number of tumor suppressor and oncogenic proteins including retinoblastoma, APC, FOXO proteins, INI1/hSNF5, galectin-3, Bok, NPM1, RASSF2, Merlin, p53, p21CIP, p27KIP1, N-WASP/FAK, estradiol receptor, Tob, BRAC1, BCR–ABL and eIF4E. Importantly, many of these proteins were found to be mislocalized in cancer cells (Hill et al., 2014). RNAi-mediated silencing of the CRM1 induced nuclear retention of p53 and cell death in cervical cancer cell lines (Santiago et al., 2013). Somatic mutations in CRM1 have been identified in chronic lymphocytic leukemia (Puente et al., 2011) and in other hematological malignancies (Sendino et al., 2018). The great majority of these mutations affect a single amino acid at position 571. The E571K mutation localizes near the NES-binding site and substitutes a glutamic acid with a lysine. This variation replaces a negatively charged residue with a positively charged amino acid and might lead to an increased affinity for NES (García-Santisteban et al., 2016).

The expression of CRM1 is increased in a broad variety of cancer types including cervical (van der Watt et al., 2009), ovarian (Noske et al., 2008), kidney (Inoue et al., 2013), lung (Gao et al., 2015) and gastric cancers (Zhou et al., 2013), as well as in glioma (Shen et al., 2009), osteosarcoma (Yao et al., 2009), esophageal carcinoma (Van Der Watt et al., 2014), hepatocellular carcinoma (Zheng et al., 2014), multiple myeloma (Schmidt et al., 2013), acute myeloid leukemia (Kojima et al., 2013), chronic myeloid/lymphoid leukemia (Lapalombella et al., 2012), mantle cell lymphoma (Yoshimura et al., 2014) and plasma cell leukemia (Tai et al., 2014). In line with these observations, high level of CRM1 expression is correlated with tumor size, the presence of distant metastasis and poor prognosis in many cancer types. Therefore, CRM1 expression might have the potential to predict clinical outcome for several human tumor types. Importantly, CRM1 also plays an important role in drug resistance (Turner and Sullivan, 2008; Turner et al., 2012). Several different nuclear export inhibitors (NEIs) have been shown to sensitize drug-resistant cancer cells to anti-cancer drugs. These data suggest that interfering with the nuclear export of tumor suppressor proteins or cell cycle inhibitors might contribute to overcome therapy resistance.

### Binding of CRM1 to Leptomycin B

**Figure 2:**
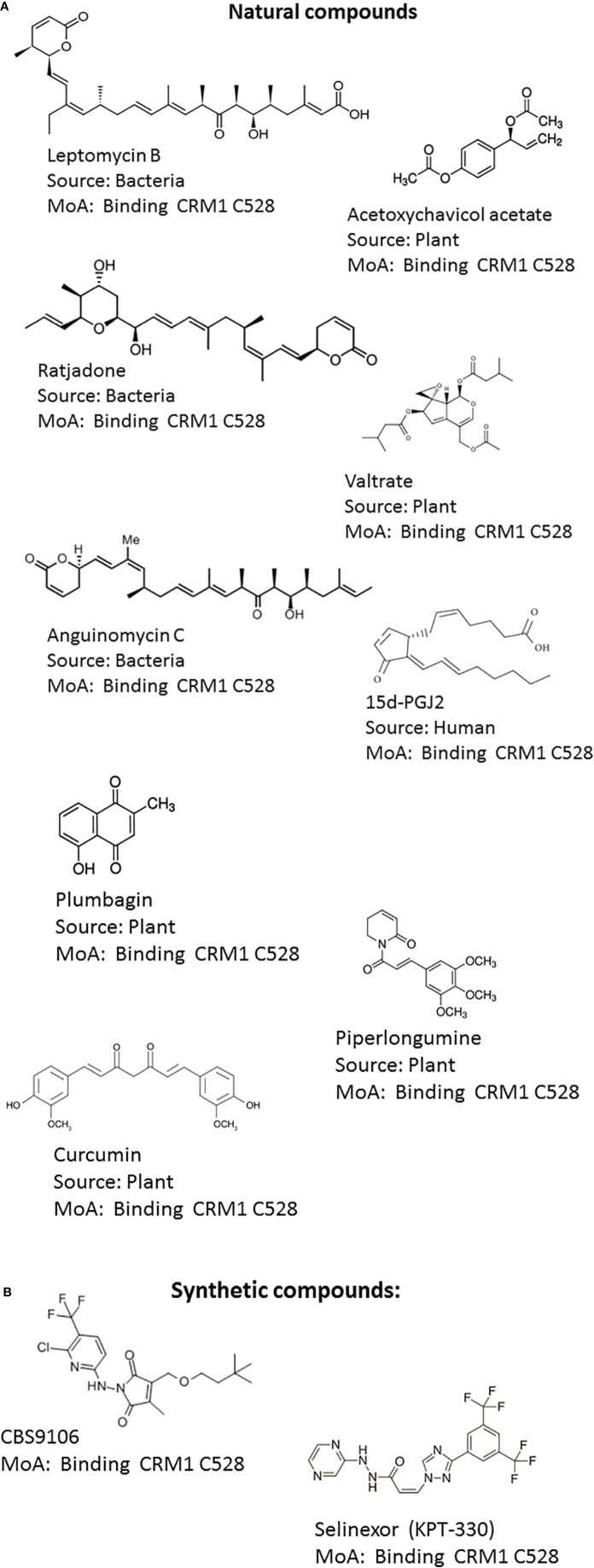
Structures of CRM1 inhibitors. **(A)** Natural compounds: (1) Leptomycin B, (2) Acetoxychavicol acetate (3) Ratjadone, (4) Valtrate, (5) Anguinomycin C, (6) 15d-PGJ2, (7) Plumbagin, (8) Curcumin (9) Piperlongumine **(B)** Synthetic compounds: (10) CBS9106, (11) KPT-330 (Selinexor).

The progress made in understanding CRM1-mediated nuclear export is greatly based on the identification of Leptomycin B (LMB) as a CRM1 inhibitor (Kudo et al., 1998). LMB is natural product polyketide isolated from Streptomyces and has been originally discovered as a potent antifungal compound (Hamamoto et al., 1983). LMB contains two conjugated dienes, an α,β-unsaturated δ-lactone, a β-hydroxy-ketone moiety, and a terminal carboxylate (**Figure 2A**). Its molecular weight is 540-Da. LMB binds covalently to a cysteine residue in CRM1 in HEAT repeat 11 of CRM1 (Cys-528 in the human CRM1) which is located in the NES-binding groove by a Michael-type addition reaction *via* its α,β-unsaturated δ-lactone moiety (Kudo et al., 1999a). As LMB modifies a cysteine residue in CRM1 critical for NES-cargo binding, it inhibits the formation of the NES–CRM1–RanGTP complex and thereby the export of the cargo protein to the cytoplasm. Surprisingly, CRM1 acts as an enzyme hydrolyzing the lactone of LMB and thereby optimizing the LMB–CRM1 interaction. CRM1-induced modification of LMB leads to the irreversibility of the conjugation (Sun et al., 2013). LMB showed promising anti-cancer activity in preclinical experiments, but failed in clinical trial due to its systemic toxicity (Newlands et al., 1996). The dose limiting toxicity associated with LMB is thought to be due to a permanent block of nuclear export of essential macromolecules.

## Targeting the CRM1-Driven Nuclear Export

Due to the crucial regulatory role and the alteration in human cancer, CRM1 has emerged as a therapeutic target for anticancer therapy. Although, altered CRM1 expression or activity is not always the driving force behind protein mislocalization, the inhibition of the nuclear export can prevent or correct aberrant subcellular protein localization (Hung and Link, 2011). For example, FOXO proteins are shuttled from the cell nucleus where they can act as tumor suppressors to the cytoplasm *via* CRM1-mediated nuclear export when they are phosphorylated by the AKT. AKT is a serine/threonine protein kinase and a key component of the PI3K/AKT signaling pathway, which is thought to be the most frequently activated signaling pathway in human cancer. While NEIs do not interfere with the signaling event that led to cytoplasmic mislocalization of FOXOs, they can trap FOXO factors in the cell nucleus and thereby promote their tumor suppressive function. Indeed, the clinically approved NEI Selinexor partially acts through trapping FOXO into the nucleus (Corno et al., 2018). Therefore, NEIs might not only be useful to treat tumors with altered CMR1 expression or function, but relocalize many tumor suppressor proteins or even mislocalize and thereby inactivate oncogenic proteins (Hung and Link, 2011). Although the groundwork to understand CRM1-mediated nuclear export has been developed over the last decades and the first generation of NEIs including LMB turned out to be to toxic to be used in the clinic, only more recently a significant therapeutic window for these inhibitors has been reported (Mutka et al., 2009). The therapeutic indications of these inhibitors are not limited to cancer but have also the potential to be used as antiviral agents.

### Natural Product and Synthetic NEIs

The known NEIs can be classified into natural products and synthetic NEIs (**Figures 2A, B**). Natural product NEIs are derived from bacterial, plant, fungal or animal sources (**Table 1**) (Sun et al., 2016). The bacterial NEIs contain a polyketide chain with a lactone ring and include LMB, anguinomycin A/B/C/D and ratjadone A/C (Hamamoto et al., 1983; Köster et al., 2003; Bonazzi et al., 2010). Anguinomycins are analogs of LMB isolated from Streptomyces sp. Ratjadone is a cytotoxin isolated from myxobacteria from soil at Cala Ratjada on Mallorca island. These polyketide natural products covalently bind to Cys-528 in the human CRM1 and have IC50 values in the low nanomolar range (Sun et al., 2013). However, these NEIs are associated with severe dose limiting toxicities. While they are very powerful tools to study CRM1 function, they are not useful as therapeutic agents. NEIs derived from plants include acetoxychavicol acetate, valtrate, piperlongumine, curcumin, dibenzylideneacetone, gonionthalamin, and plumbagin. They are thought to bind to Cys528 of CRM1 with low affinity and inhibit CRM1 in the micromolar range. Acetoxychavicol acetate (ACA) is found in *Alpinia galangal* and was identified as an inhibitor of CRM1-dependent nuclear export by screening extracts from more than 600 medical plants (Ye and Li, 2006). Pharmacophore features of ACA have been defined. Similar to ACA, the chemically unrelated valtrate was found through screening 200 plant extracts. Valtrate is an iridoid ester with moderate lipophilicity extracted from *Valerianae radix* (Murakami et al., 2002). Both, ACA and valtrate have been shown to inhibit virus production (Watanabe et al., 2011). Furthermore, two food additives, namely piperlongumine and curcumin were found to inhibit CRM1-mediated nuclear export. The alkaloid piperlongumine is extracted from *Piper longum* Linn and exhibits multiple beneficial effects including selective cytotoxicity against several cancer cell lines. Accordingly, piperlongumine was shown to inhibit the CRM1 dependent transport of tumor suppressor proteins including FOXO1 and p21 (Niu et al., 2015b). Curcumin is a polyphenol compound present in *Curcuma longa* plant and is the main component of the Indian spice turmeric. It is known for its antioxidant, anti‐cancer and anti‐inflammatory effects. Curcumin and its structural analogue dibenzylideneacetone were shown to prevent the cytoplasmic accumulation of a reporter protein fused with NES in a CRM1-dependent manner (Niu et al., 2013). Similarly, a nuclear export assay and molecular modeling were used to characterize the styryl-lactone compound goniothalamin present in *Goniothalamus macrophyllus* as an inhibitor of nucleocytoplasmic transport *via* CRM1 (Wach et al., 2010). The natural bicyclic naphthoquinone plumbagin found in roots of plumbaginaceae which have been used in Indian traditional medicine was also identified as a CRM1 inhibitor and capable of blocking the nuclear export of RanBP1 and FOXO1 (Liu et al., 2014). Interestingly, the endogenous, anti-inflammatory prostaglandin 15d-PGJ2 has been shown to inhibit CRM1 by a LMB-like mechanism but with much less potency (Hilliard et al., 2010). Conversely, the mechanism of action for the diterpenoid oridonin present in *Rabdosia rubescens* remains to be determined. Oridonin inhibits inflammation and carcinogenesis and has been used in Chinese traditional medicine. It was shown to induce nuclear translocation of Crm1 and to increase the expression and nuclear accumulation of nucleoporin98, involved in Crm1-mediated nuclear export (Li et al., 2014).

**Table 1:** Natural product nuclear export inhibitors.

Natural Product	Source	Mode of Action	Effect
Leptomycin B	Streptomyces	CRM1-Cys528	Potent nuclear export inhibition
Anguinomycin	Streptomyces	CRM1-Cys528	Potent nuclear export inhibition
Ratjadone	Myxobacteria	CRM1-Cys528	Potent nuclear export inhibition
Acetoxychavicol acetate	Alpinia galangal	CRM1-Cys528	Inhibit viral production
Valtrate	Valerianae Radix	CRM1-Cys528	Inhibit viral production
Piperlongumine	Piper longum	CRM1-Cys528	Nuclear tumor suppressor proteins
Curcumin	Curcuma longa	CRM1-Cys528	Nuclear reporter protein
Gonionthalamin	Goniothalamus macrophyllus	CRM1-Cys528	Nuclear reporter protein
Plumbagin	Plumbaginaceae	CRM1-Cys528	Nuclear RanBP1 and FOXO1
15d-PGJ2	Human prostaglandine	CRM1-Cys528	Nuclear TFIIAα
Oridonin	Rabdosia rubescens	n/d	Increased and nuclear Nup98
MDN-0105	Fungal metabolite	CRM1 n/d	Nuclear FOXO3, Rev, NF-κB

The failure of the first generation of NEIs in clinical trials raised skepticism about the therapeutic potential of inhibiting nuclear export. Furthermore, preclinical evaluation of the semi-synthetic LMB derivative KOS-2462 (Mutka et al., 2009) and the synthetic small molecule CRM1 inhibitor CBS9106 (Sakakibara et al., 2011) did not lead to their clinical development (Parikh et al., 2014). The realization that there is the possibility of targeting nuclear export with an acceptable level of toxicity have spurred the development of second generation, synthetic CRM1 inhibitors, including CBS9106 (Sakakibara et al., 2011), PKF050-638 (Daelemans et al., 2002), 5219668 (Kau et al., 2003), compound3/4 (Monovich et al., 2009) and S109 (Niu et al., 2015a) and Selective Inhibitors of Nuclear Export (SINEs) (Kalid et al., 2012; Lapalombella et al., 2012; Ranganathan et al., 2012). The first molecule of this family of small molecule compounds was found by a screening effort aimed to identify new anti-HIV Rev inhibitors (Daelemans et al., 2002). The authors analyzed a collection of small molecules using a Rev-dependent luciferase reporter assay and identified PKF050-638 as an anti-viral agent. The SINE compounds include selinexor (KPT-330), verdinexor (KPT-335), KPT-185, KPT-276, and KPT-251. SINEs covalently bind to Cysteine 528 residue in a slowly reversible fashion. While a homozygous or heterozygous mutation of Cys528 conferred resistance to SINE treatment, the E571K mutation did not affect their inhibitory efficacy on the CRM1-mediated nuclear export (García-Santisteban et al., 2016; Jardin et al., 2016). Although, LMB and SINE share the same target amino acid residue of CRM1, SINEs are smaller and occupy less space of the NES groove (Lapalombella et al., 2012; Etchin et al., 2013; Haines et al., 2015; Hing et al., 2016). In addition, SINEs don´t undergo hydrolysis upon binding to CRM1 and don´t form a salt bridge with CRM1 (Etchin et al., 2013; Sun et al., 2013; Haines et al., 2015). Therefore, the binding of SINEs to CRM1 is slowly reversible (Sun et al., 2013). As a consequence inhibition of CRM1 mediated nuclear export is transient which might explain the reduced toxicity of SINEs compared to the one associated with LMB. SINE compounds have been analyzed in preclinical and clinical studies for numerous solid and hematologic cancers. These clinical trials include the treatment of lymphomas (non-Hodgkin’s and diffuse large B-cell lymphoma), gliomas, sarcomas, breast cancer, lung cancer, pancreatic cancer, Myelodysplastic Syndromes (MDS), acute myeloid leukemia (AML), Acute lymphocytic leukemia (ALL) multiple myeloma, gastric cancer, esophageal cancer, colorectal cancer, prostate cancer, melanoma, thymic cancer, and gynecologic cancers (Wang and Liu, 2019). The spectrum of malignancies evaluated in these clinical trials underlines the broad applicability of CRM1 inhibitors (Wang and Liu, 2019). The recent clinical approval of Selinexor (Xpovio) also known as KPT-330 is the proof of concept for the therapeutic utility of manipulating the nuclear export. Selinexor has been approved for the treatment of patients with relapsed refractory multiple myeloma (Chari et al., 2019). Side effects associated with SINE treatment are less severe as expected from agents capable of inhibiting a core physiological process as the nuclear export of macromolecule.

## Natural Products as a Source of Novel CRM1 Inhibitors

Natural products are considered as an extremely valuable source for the discovery of new drugs against diverse pathologies. As yet only a fraction of the diversity of bioactive compounds has been explored and opportunities for discovering new natural products leading to new drugs are huge. Several approaches for identifying CRM1 Inhibitors have been reported (**Box 1**). Bacterial CRM1 inhibitors like LMB might provide Streptomyces with the capacity to kill fungi which restrict their growth. CRM1 is essential for the fungus. A change from Cys529 to Ser in a CRM1 mutant of *Saccharomyces pombe* render the fungus resistant to LMB, whereas Saccharomyces cerevisiae which does not carry a cysteine at that position is LMB insensitive (Kudo et al., 1999b; Yashiroda and Yoshida, 2003). Many organisms might have developed similar or slightly different capacities to fight and resist competitors. Therefore, is not fare-fetched to expect metabolites from natural origin to represent a rich source of potential nuclear export inhibitors with novel modes of action. Mutating Cys528 is an obvious yet not evaluated possibility of cancer cells to escape the selective pressure imposed by the treatment with covalent CRM1 inhibitors. It is also possible, that the therapeutic efficacy of CRM1 inhibition might also be limited in cancer cells that strongly overexpress CRM1, but increasing the dosing of covalent CRM1 inhibitors is impossible due to on-target and off-target toxicity. For the time being, all known CRM1 inhibitors act through covalent binding to Cys528 of CRM1. In addition, they all contain a Michael acceptor which makes them prone to target off effects by non-specific interaction with other targets. The perspective to identify and develop non-covalent inhibitors of CRM1 holds promise to increase efficacy by reducing drug resistance and on-target and off-target toxicity.

Box 1 **Approaches for identifying CRM1 Inhibitors.**

**Approaches for identifying CRM1 Inhibitors**

Several methods are useful in identifying CRM1 inhibitors including gene reporter assays and image-based high content screening.

**Gene reporter assay**

Daelemans et al. reported a screening campaign to identify inhibitors of the CRM1-mediated nuclear export of HIV-1 Rev protein using Rev-dependent luciferase reporter gene assay in Jurkat T cells (Daelemans et al., 2002). The reporter gene is flanked by splice sites under the control of the HIV-1 Rev response element (RRE). The cells are co-transfected firefly luciferase reporter gene fused to the p17 gag sequences and the RRE, flanked by the HIV-1 major splice sites, and driven by the CMV promoter and a vector that expresses HIV-1 Rev protein. Inhibitors of the Rev function cause a dose-dependent inhibition of Rev-dependent luciferase expression. The specificity of the compounds on Rev function can be tested on a Rev independent luciferase gene.

**High Content Screening (HCS)**

Image-based HCS (Zanella et al., 2010) has been widely used to screen for CRM1 inhibitors. The U2nesRELOC system uses human U2OS osteosarcoma cells that stably express a green fluorescent protein (GFP)-labeled Rev reporter protein that contains a heterologous nuclear export signal (NES). The fluorescent signal of U2nesRELOC cells is exclusively expressed in the cytoplasm. Treatment with NEIs such as LMB the fluorescent reporter rapidly accumulates in the nucleus (Cautain et al., 2015a). Acquisition and analysis of images is based on high content screening technology (Zanella et al., 2010). LMB is used as a positive control. As the cell nucleus is stained with 4,6-diamidino-2-phenylindole (DAPI), the cell nucleus can be visualized and the definition of its boundaries can support automated image analysis (Machado et al., 2019). In order to identify small molecule agents that inhibit the nuclear export of tumor suppressors from the nucleus *via* CRM1, we used the U2nesRELOC system to screen a library of natural product extracts from microbial origin. The collection of products was enriched in secondary metabolites (Cautain et al., 2014). 14,000 different extracts were evaluated for the capacity to accumulate the fluorescent signal in the nucleus of the reporter cells. From the 14,000 extracts, 6000 were obtained from fungi, another 6000 were derived from actinomycetes, and the remaining 2000 extracts were of marine actinomycete origin. 12 extracts with nuclear export inhibitory activities that were not associated with previously known active metabolites were identified. After purification of active compounds, several chemical structures of novel nuclear export inhibitors were identified including the fungal metabolite MDN-0105.

Natural product drug discovery is associated with specific limitations including the complexity of natural product chemistry, intellectual property landscape and sustainable supply. Bioavailability challenges associated with natural products could potentially be addressed by designing suitable drug delivery strategies.

## Concluding Remarks

Interrupting the nuclear export of viral and cellular proteins mediated by CRM1 has emerged as an extremely promising therapeutic strategy to treat patients with viral infections and cancer. The clinical approval of the first-in-class CRM1 inhibitor Selinexor has proven the therapeutic potential of CRM1 inhibition for the treatment of cancer. Compounds derived from natural sources hold promise to support the discovery of new NEIs with a novel mode of action. Image-based high content screening of extract and compound collections is a very efficient way to identify natural products with inhibitory effect on the nuclear export.

## Author Contributions

All authors contributed to the critical discussion, text and figure preparation, and proofreading of the current manuscript.

## Funding

This work was supported by Fundação para a Ciência e a Tecnologia (FCT) Research Center Grant UID/BIM/04773/2013 Centre for Biomedical Research 1334 and by the Spanish Ministry of Science, Innovation and Universities through Grant RTI2018-094629-B-I00 to WL. BF was supported by FCT-SFRH/BPD/100434/2014 and Marie Curie Individual Fellowship project TRIBBLES (#748585). This work was also supported by two LPCC-NRS/Terry Fox grants (2016/2017; 2017/2018).

## Conflict of Interest

The authors declare that the research was conducted in the absence of any commercial or financial relationships that could be construed as a potential conflict of interest.
